# Bis(2-amino­pyrazine-κ*N* ^1^)tetra­aqua­cadmium(II) bis­(perchlorate)–2-amino­pyrazine (1/4)

**DOI:** 10.1107/S1600536809048387

**Published:** 2009-11-21

**Authors:** Xiao-Li Cheng, Shan Gao, Seik Weng Ng

**Affiliations:** a College of Chemistry and Materials Science, Heilongjiang University, Harbin 150080, People’s Republic of China; b Department of Chemistry, University of Malaya, 50603 Kuala Lumpur, Malaysia

## Abstract

In the title compound, [Cd(C_4_H_5_N_3_)_2_(H_2_O)_4_](ClO_4_)_2_·4C_4_H_5_N_3_, the Cd^II^ atom (site symmetry 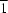) is coordinated by two *N*-heterocycles and four water mol­ecules, resulting in a distorted *trans*-CdN_2_O_4_ octa­hedral geometry for the metal. In the crystal, the cation, anion and free *N*-heterocycle mol­ecules are linked by N—H⋯N, N—H⋯O, O—H⋯N and O—H⋯O hydrogen bonds, forming a three-dimensional network.

## Related literature

For the cadmium nitrate adduct of 2-amino­pyrazine, see: Tai *et al.* (2008 ▶).
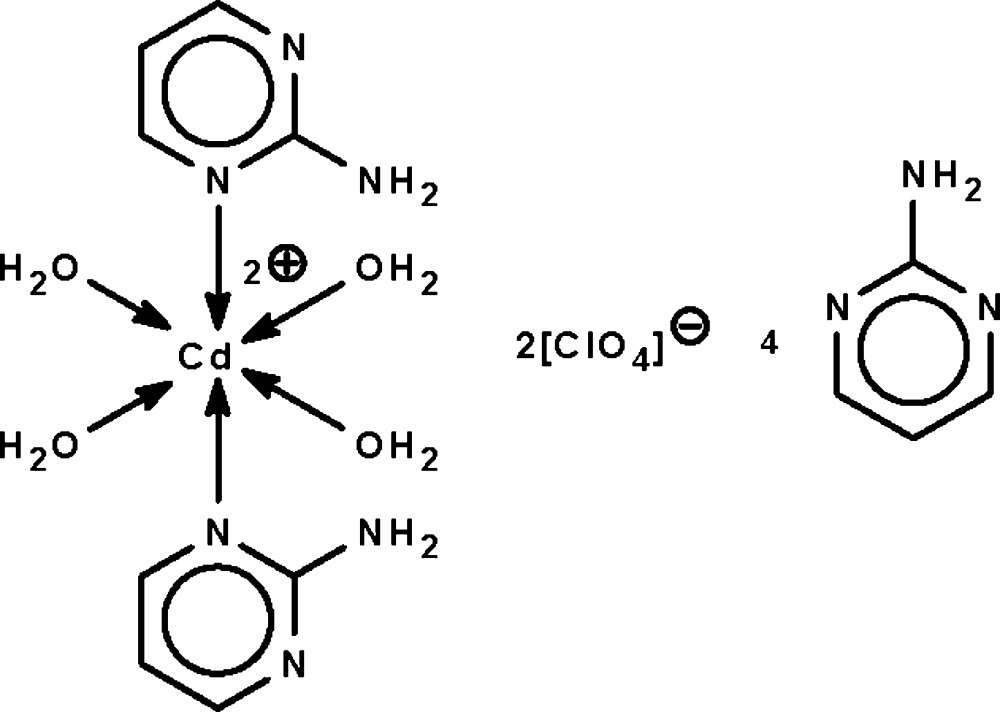


## Experimental

### 

#### Crystal data

[Cd(C_4_H_5_N_3_)_2_(H_2_O)_4_](ClO_4_)_2_·4C_4_H_5_N_3_
*M* *_r_* = 954.02
Monoclinic, 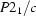

*a* = 8.8912 (2) Å
*b* = 23.2402 (4) Å
*c* = 9.3689 (2) Å
β = 96.4263 (7)°
*V* = 1923.76 (7) Å^3^
*Z* = 2
Mo *K*α radiation
μ = 0.79 mm^−1^
*T* = 293 K
0.18 × 0.15 × 0.15 mm

#### Data collection

Rigaku R-AXIS RAPID IP diffractometer
Absorption correction: multi-scan (*ABSCOR*; Higashi, 1995 ▶) *T*_min_ = 0.871, *T*_max_ = 0.891
18645 measured reflections
4393 independent reflections
3982 reflections with *I* > 2σ(*I*)
*R*_int_ = 0.024

#### Refinement

*R*[*F* ^2^ > 2σ(*F* ^2^)] = 0.026
*wR*(*F* ^2^) = 0.072
*S* = 1.08
4393 reflections
299 parameters
10 restraints
H atoms treated by a mixture of independent and constrained refinement
Δρ_max_ = 0.34 e Å^−3^
Δρ_min_ = −0.58 e Å^−3^

### 

Data collection: *RAPID-AUTO* (Rigaku, 1998 ▶); cell refinement: *RAPID-AUTO*; data reduction: *CrystalClear* (Rigaku/MSC, 2002 ▶); program(s) used to solve structure: *SHELXS97* (Sheldrick, 2008 ▶); program(s) used to refine structure: *SHELXL97* (Sheldrick, 2008 ▶); molecular graphics: *X-SEED* (Barbour, 2001 ▶); software used to prepare material for publication: *publCIF* (Westrip, 2009 ▶).

## Supplementary Material

Crystal structure: contains datablocks global, I. DOI: 10.1107/S1600536809048387/hb5230sup1.cif

Structure factors: contains datablocks I. DOI: 10.1107/S1600536809048387/hb5230Isup2.hkl

Additional supplementary materials:  crystallographic information; 3D view; checkCIF report

## Figures and Tables

**Table 1:** Selected bond lengths (Å)

Cd1—O1*W*	2.282 (1)
Cd1—O2*W*	2.367 (1)
Cd1—N1	2.323 (1)

**Table 2:** Hydrogen-bond geometry (Å, °)

*D*—H⋯*A*	*D*—H	H⋯*A*	*D*⋯*A*	*D*—H⋯*A*
O1w—H11⋯N4	0.84 (1)	1.92 (1)	2.758 (2)	171 (3)
O1w—H12⋯N6^i^	0.84 (1)	2.24 (1)	3.059 (3)	165 (2)
O2w—H21⋯N7	0.84 (1)	1.92 (1)	2.756 (2)	178 (3)
O2w—H22⋯O1	0.84 (1)	1.98 (1)	2.806 (2)	167 (3)
N3—H31⋯O2w^ii^	0.85 (1)	2.28 (1)	3.070 (2)	154 (2)
N3—H32⋯N5^iii^	0.85 (1)	2.28 (1)	3.127 (2)	175 (2)
N6—H61⋯N2^iii^	0.85 (1)	2.23 (1)	3.071 (2)	173 (2)
N6—H62⋯O2^i^	0.85 (1)	2.35 (1)	3.140 (2)	155 (2)
N9—H91⋯O3^iv^	0.85 (1)	2.20 (1)	3.009 (4)	159 (3)
N9—H92⋯O4	0.85 (1)	2.41 (2)	3.073 (3)	135 (3)

Symmetry codes: (i) 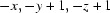; (ii) 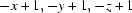; (iii) 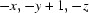; (iv) 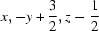.

**Fig. 1.**
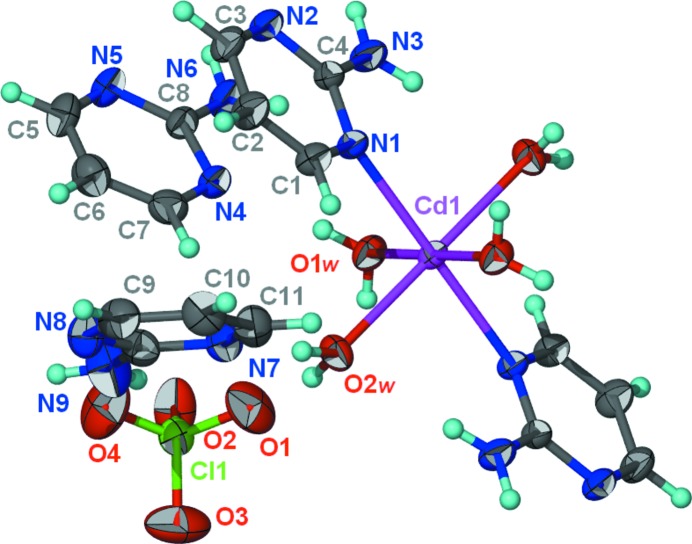
The molecular structure of (I) shown at the 50% probability level; hydrogen atoms are drawn as spheres of arbitrary radius. Unlabelled atoms are generated by the symmetry operation (1–x, 1–y, 1–z).

## supplementary crystallographic information

### Experimental

To an aqueous solution of 2-aminopyrimidine (0.19 g, 2 mmol) was added cadmium perchlorate hydrate (0.662 g, 2 mmol). Colorless prisms of (I) separated from the solution after a few days.

### Refinement

Carbon-bound H-atoms were placed in calculated positions (C—H = 0.93 Å) and were included in the refinement in the riding model approximation, with *U*_iso_(H) = 1.2*U*_eq_(C). The amino and water H-atoms were located in a difference Fourier map, and were refined with a distance restraint of N–H = O–H = 0.85±0.01 Å; their U_iso_ values were refined.

### Crystal data

**Table:**

[Cd(C_4_H_5_N_3_)_2_(H_2_O)_4_](ClO_4_)_2_·4C_4_H_5_N_3_	*F*(000) = 972
*M**_r_* = 954.02	*D*_x_ = 1.647 Mg m^−^^3^
Monoclinic, *P*2_1_/*c*	Mo *K*α radiation, λ = 0.71073 Å
Hall symbol: -P 2ybc	Cell parameters from 16676 reflections
*a* = 8.8912 (2) Å	θ = 3.0–27.5°
*b* = 23.2402 (4) Å	µ = 0.79 mm^−^^1^
*c* = 9.3689 (2) Å	*T* = 293 K
β = 96.4263 (7)°	Prism, colorless
*V* = 1923.76 (7) Å^3^	0.18 × 0.15 × 0.15 mm
*Z* = 2	

### Data collection

**Table:**

Rigaku R-AXIS RAPID IP diffractometer	4393 independent reflections
Radiation source: fine-focus sealed tube	3982 reflections with *I* > 2σ(*I*)
graphite	*R*_int_ = 0.024
ω scan	θ_max_ = 27.5°, θ_min_ = 3.1°
Absorption correction: multi-scan (*ABSCOR*; Higashi, 1995)	*h* = −11→11
*T*_min_ = 0.871, *T*_max_ = 0.891	*k* = −30→30
18645 measured reflections	*l* = −12→12

### Refinement

**Table:**

Refinement on *F*^2^	Primary atom site location: structure-invariant direct methods
Least-squares matrix: full	Secondary atom site location: difference Fourier map
*R*[*F*^2^ > 2σ(*F*^2^)] = 0.026	Hydrogen site location: inferred from neighbouring sites
*wR*(*F*^2^) = 0.072	H atoms treated by a mixture of independent and constrained refinement
*S* = 1.08	*w* = 1/[σ^2^(*F*_o_^2^) + (0.041*P*)^2^ + 0.6531*P*] where *P* = (*F*_o_^2^ + 2*F*_c_^2^)/3
4393 reflections	(Δ/σ)_max_ = 0.001
299 parameters	Δρ_max_ = 0.34 e Å^−^^3^
10 restraints	Δρ_min_ = −0.58 e Å^−^^3^

### Fractional atomic coordinates and isotropic or equivalent isotropic displacement parameters (Å^2^)

**Table:**

	*x*	*y*	*z*	*U*_iso_*/*U*_eq_	
Cd1	0.5000	0.5000	0.5000	0.02847 (7)	
Cl1	0.09644 (5)	0.653770 (19)	0.68436 (5)	0.04031 (11)	
O1	0.2212 (2)	0.61643 (9)	0.6674 (2)	0.0791 (6)	
O2	−0.03933 (19)	0.62083 (7)	0.6713 (2)	0.0740 (5)	
O3	0.1197 (3)	0.67800 (11)	0.8235 (2)	0.0915 (7)	
O4	0.0861 (3)	0.69789 (9)	0.5810 (2)	0.0915 (7)	
O1W	0.24457 (16)	0.49525 (6)	0.50677 (16)	0.0390 (3)	
H11	0.191 (3)	0.5138 (10)	0.443 (2)	0.058 (7)*	
H12	0.214 (3)	0.5019 (9)	0.5870 (18)	0.057 (8)*	
O2W	0.50795 (16)	0.59683 (5)	0.57870 (14)	0.0396 (3)	
H21	0.534 (3)	0.6274 (7)	0.541 (3)	0.067 (8)*	
H22	0.4296 (19)	0.6048 (11)	0.618 (3)	0.064 (8)*	
N1	0.46982 (16)	0.52799 (6)	0.26042 (14)	0.0307 (3)	
N2	0.33224 (18)	0.53698 (7)	0.02729 (15)	0.0390 (3)	
N3	0.29425 (19)	0.45974 (7)	0.17037 (16)	0.0410 (3)	
H31	0.322 (3)	0.4384 (8)	0.2423 (18)	0.052 (6)*	
H32	0.235 (2)	0.4467 (9)	0.1011 (18)	0.047 (6)*	
N4	0.09370 (18)	0.56440 (7)	0.29843 (16)	0.0388 (3)	
N5	−0.08831 (18)	0.58548 (7)	0.09866 (17)	0.0442 (4)	
N6	−0.0911 (2)	0.49855 (7)	0.2172 (2)	0.0469 (4)	
H61	−0.151 (2)	0.4875 (10)	0.1456 (19)	0.048 (6)*	
H62	−0.031 (2)	0.4731 (8)	0.256 (3)	0.055 (7)*	
N7	0.5948 (2)	0.69581 (7)	0.44802 (18)	0.0450 (4)	
N8	0.5465 (2)	0.78730 (7)	0.3350 (2)	0.0517 (4)	
N9	0.3655 (3)	0.74176 (11)	0.4486 (3)	0.0756 (7)	
H91	0.304 (3)	0.7695 (9)	0.432 (3)	0.077 (9)*	
H92	0.329 (3)	0.7163 (10)	0.500 (3)	0.079 (9)*	
C1	0.5453 (2)	0.57603 (8)	0.23394 (18)	0.0374 (4)	
H1	0.6196	0.5892	0.3041	0.045*	
C2	0.5189 (2)	0.60663 (8)	0.1092 (2)	0.0445 (4)	
H2	0.5728	0.6398	0.0927	0.053*	
C3	0.4069 (2)	0.58525 (9)	0.00885 (19)	0.0436 (4)	
H3	0.3827	0.6058	−0.0758	0.052*	
C4	0.3672 (2)	0.50879 (7)	0.15276 (18)	0.0314 (3)	
C5	−0.0272 (3)	0.63751 (9)	0.0928 (2)	0.0507 (5)	
H5	−0.0691	0.6630	0.0228	0.061*	
C6	0.0952 (3)	0.65560 (9)	0.1854 (2)	0.0514 (5)	
H6	0.1368	0.6921	0.1791	0.062*	
C7	0.1519 (2)	0.61674 (9)	0.2871 (2)	0.0456 (4)	
H7	0.2347	0.6274	0.3512	0.055*	
C8	−0.02538 (19)	0.55082 (8)	0.20305 (18)	0.0351 (3)	
C9	0.6867 (3)	0.78674 (9)	0.3004 (2)	0.0515 (5)	
H9	0.7190	0.8176	0.2485	0.062*	
C10	0.7873 (3)	0.74272 (10)	0.3375 (3)	0.0541 (5)	
H10	0.8859	0.7435	0.3134	0.065*	
C11	0.7340 (2)	0.69784 (9)	0.4116 (2)	0.0506 (5)	
H11A	0.7989	0.6673	0.4377	0.061*	
C12	0.5058 (2)	0.74143 (8)	0.4095 (2)	0.0443 (4)	

### Atomic displacement parameters (Å^2^)

**Table:**

	*U*^11^	*U*^22^	*U*^33^	*U*^12^	*U*^13^	*U*^23^
Cd1	0.03046 (10)	0.03085 (10)	0.02343 (9)	−0.00094 (6)	0.00002 (6)	0.00250 (5)
Cl1	0.0351 (2)	0.0404 (2)	0.0463 (2)	0.00001 (17)	0.00821 (17)	0.00029 (18)
O1	0.0470 (9)	0.0858 (13)	0.1065 (15)	0.0166 (9)	0.0179 (9)	−0.0229 (11)
O2	0.0410 (8)	0.0577 (10)	0.1204 (16)	−0.0111 (7)	−0.0042 (9)	0.0201 (10)
O3	0.0815 (14)	0.1346 (19)	0.0612 (11)	−0.0055 (13)	0.0194 (10)	−0.0369 (12)
O4	0.1003 (16)	0.0766 (13)	0.0950 (15)	−0.0187 (11)	−0.0007 (12)	0.0428 (11)
O1W	0.0313 (6)	0.0494 (8)	0.0356 (7)	0.0028 (5)	0.0013 (5)	0.0050 (5)
O2W	0.0476 (7)	0.0292 (6)	0.0440 (7)	0.0020 (5)	0.0133 (6)	0.0006 (5)
N1	0.0335 (7)	0.0336 (7)	0.0243 (6)	−0.0003 (5)	0.0007 (5)	0.0016 (5)
N2	0.0413 (8)	0.0463 (8)	0.0276 (6)	0.0034 (6)	−0.0034 (6)	0.0030 (6)
N3	0.0487 (9)	0.0396 (8)	0.0320 (7)	−0.0089 (7)	−0.0069 (7)	0.0004 (6)
N4	0.0375 (8)	0.0432 (8)	0.0338 (7)	0.0055 (6)	−0.0039 (6)	−0.0007 (6)
N5	0.0398 (8)	0.0505 (9)	0.0401 (8)	0.0005 (7)	−0.0047 (6)	0.0119 (7)
N6	0.0480 (10)	0.0432 (9)	0.0463 (10)	−0.0037 (7)	−0.0091 (8)	0.0079 (7)
N7	0.0522 (10)	0.0364 (8)	0.0461 (9)	−0.0054 (7)	0.0046 (7)	0.0029 (7)
N8	0.0538 (10)	0.0419 (9)	0.0593 (10)	−0.0003 (8)	0.0061 (8)	0.0089 (8)
N9	0.0579 (13)	0.0616 (14)	0.113 (2)	0.0022 (11)	0.0351 (13)	0.0136 (13)
C1	0.0378 (9)	0.0410 (9)	0.0325 (8)	−0.0051 (7)	0.0009 (7)	0.0019 (7)
C2	0.0506 (11)	0.0431 (10)	0.0397 (9)	−0.0076 (8)	0.0054 (8)	0.0101 (8)
C3	0.0505 (11)	0.0491 (10)	0.0307 (8)	0.0048 (8)	0.0027 (7)	0.0111 (7)
C4	0.0327 (8)	0.0350 (8)	0.0264 (7)	0.0046 (6)	0.0022 (6)	−0.0012 (6)
C5	0.0529 (12)	0.0493 (11)	0.0491 (11)	0.0058 (9)	0.0018 (9)	0.0169 (9)
C6	0.0549 (12)	0.0396 (10)	0.0602 (12)	−0.0035 (9)	0.0084 (10)	0.0019 (9)
C7	0.0423 (10)	0.0480 (10)	0.0455 (10)	−0.0015 (8)	0.0006 (8)	−0.0093 (8)
C8	0.0323 (8)	0.0416 (9)	0.0311 (8)	0.0040 (7)	0.0022 (6)	0.0019 (7)
C9	0.0599 (13)	0.0458 (10)	0.0491 (11)	−0.0110 (9)	0.0079 (9)	0.0050 (9)
C10	0.0427 (11)	0.0554 (12)	0.0649 (13)	−0.0085 (9)	0.0098 (10)	−0.0018 (10)
C11	0.0462 (11)	0.0441 (10)	0.0597 (12)	0.0000 (8)	−0.0018 (9)	−0.0013 (9)
C12	0.0478 (10)	0.0378 (9)	0.0475 (10)	−0.0050 (8)	0.0061 (8)	−0.0028 (8)

### Geometric parameters (Å, °)

**Table:**

Cd1—O1W	2.282 (1)	N6—C8	1.361 (2)
Cd1—O2W	2.367 (1)	N6—H61	0.85 (1)
Cd1—O1W^i^	2.282 (1)	N6—H62	0.85 (1)
Cd1—N1^i^	2.323 (1)	N7—C11	1.321 (3)
Cd1—N1	2.323 (1)	N7—C12	1.348 (3)
Cd1—O2W^i^	2.367 (1)	N8—C9	1.323 (3)
Cl1—O4	1.406 (2)	N8—C12	1.345 (3)
Cl1—O3	1.414 (2)	N9—C12	1.338 (3)
Cl1—O2	1.423 (2)	N9—H91	0.85 (1)
Cl1—O1	1.431 (2)	N9—H92	0.85 (1)
O1W—H11	0.84 (1)	C1—C2	1.366 (2)
O1W—H12	0.84 (1)	C1—H1	0.9300
O2W—H21	0.84 (1)	C2—C3	1.383 (3)
O2W—H22	0.84 (1)	C2—H2	0.9300
N1—C1	1.340 (2)	C3—H3	0.9300
N1—C4	1.358 (2)	C5—C6	1.380 (3)
N2—C3	1.324 (3)	C5—H5	0.9300
N2—C4	1.351 (2)	C6—C7	1.367 (3)
N3—C4	1.331 (2)	C6—H6	0.9300
N3—H31	0.85 (1)	C7—H7	0.9300
N3—H32	0.85 (1)	C9—C10	1.378 (3)
N4—C7	1.331 (3)	C9—H9	0.9300
N4—C8	1.344 (2)	C10—C11	1.367 (3)
N5—C5	1.329 (3)	C10—H10	0.9300
N5—C8	1.340 (2)	C11—H11A	0.9300
			
O1W—Cd1—O1W^i^	180.0	C9—N8—C12	115.77 (18)
O1W—Cd1—N1^i^	88.13 (5)	C12—N9—H91	124 (2)
O1W^i^—Cd1—N1^i^	91.87 (5)	C12—N9—H92	125 (2)
O1W—Cd1—N1	91.87 (5)	H91—N9—H92	111 (3)
O1W^i^—Cd1—N1	88.13 (5)	N1—C1—C2	123.39 (16)
N1^i^—Cd1—N1	180.0	N1—C1—H1	118.3
O1W—Cd1—O2W^i^	88.14 (5)	C2—C1—H1	118.3
O1W^i^—Cd1—O2W^i^	91.86 (5)	C1—C2—C3	115.88 (17)
N1^i^—Cd1—O2W^i^	91.78 (5)	C1—C2—H2	122.1
N1—Cd1—O2W^i^	88.22 (5)	C3—C2—H2	122.1
O1W—Cd1—O2W	91.86 (5)	N2—C3—C2	123.28 (16)
O1W^i^—Cd1—O2W	88.14 (5)	N2—C3—H3	118.4
N1^i^—Cd1—O2W	88.22 (5)	C2—C3—H3	118.4
N1—Cd1—O2W	91.78 (5)	N3—C4—N2	117.09 (16)
O2W^i^—Cd1—O2W	180.0	N3—C4—N1	119.05 (15)
O4—Cl1—O3	109.65 (15)	N2—C4—N1	123.85 (16)
O4—Cl1—O2	110.08 (12)	N5—C5—C6	123.28 (18)
O3—Cl1—O2	109.20 (14)	N5—C5—H5	118.4
O4—Cl1—O1	110.95 (15)	C6—C5—H5	118.4
O3—Cl1—O1	107.97 (14)	C7—C6—C5	116.11 (19)
O2—Cl1—O1	108.96 (12)	C7—C6—H6	121.9
Cd1—O1W—H11	116.4 (19)	C5—C6—H6	121.9
Cd1—O1W—H12	116 (2)	N4—C7—C6	122.90 (18)
H11—O1W—H12	109 (3)	N4—C7—H7	118.5
Cd1—O2W—H21	132.5 (19)	C6—C7—H7	118.5
Cd1—O2W—H22	110.3 (18)	N5—C8—N4	125.27 (17)
H21—O2W—H22	106 (3)	N5—C8—N6	117.33 (16)
C1—N1—C4	116.43 (14)	N4—C8—N6	117.34 (16)
C1—N1—Cd1	113.95 (10)	N8—C9—C10	123.3 (2)
C4—N1—Cd1	128.40 (11)	N8—C9—H9	118.4
C3—N2—C4	117.02 (15)	C10—C9—H9	118.4
C4—N3—H31	119.6 (16)	C11—C10—C9	116.3 (2)
C4—N3—H32	119.0 (15)	C11—C10—H10	121.9
H31—N3—H32	120 (2)	C9—C10—H10	121.9
C7—N4—C8	116.48 (16)	N7—C11—C10	123.1 (2)
C5—N5—C8	115.96 (16)	N7—C11—H11A	118.4
C8—N6—H61	115.5 (17)	C10—C11—H11A	118.4
C8—N6—H62	114.2 (18)	N9—C12—N8	116.8 (2)
H61—N6—H62	116 (2)	N9—C12—N7	117.91 (19)
C11—N7—C12	116.22 (17)	N8—C12—N7	125.3 (2)
			
O1W—Cd1—N1—C1	127.25 (12)	Cd1—N1—C4—N2	162.47 (13)
O1W^i^—Cd1—N1—C1	−52.75 (12)	C8—N5—C5—C6	1.1 (3)
O2W^i^—Cd1—N1—C1	−144.67 (12)	N5—C5—C6—C7	−0.5 (3)
O2W—Cd1—N1—C1	35.33 (12)	C8—N4—C7—C6	0.4 (3)
O1W—Cd1—N1—C4	−39.56 (14)	C5—C6—C7—N4	−0.3 (3)
O1W^i^—Cd1—N1—C4	140.44 (14)	C5—N5—C8—N4	−1.1 (3)
O2W^i^—Cd1—N1—C4	48.52 (14)	C5—N5—C8—N6	176.16 (19)
O2W—Cd1—N1—C4	−131.48 (14)	C7—N4—C8—N5	0.3 (3)
C4—N1—C1—C2	2.8 (3)	C7—N4—C8—N6	−176.88 (18)
Cd1—N1—C1—C2	−165.63 (16)	C12—N8—C9—C10	−0.3 (3)
N1—C1—C2—C3	0.3 (3)	N8—C9—C10—C11	1.1 (3)
C4—N2—C3—C2	1.5 (3)	C12—N7—C11—C10	−0.8 (3)
C1—C2—C3—N2	−2.6 (3)	C9—C10—C11—N7	−0.4 (3)
C3—N2—C4—N3	−178.82 (18)	C9—N8—C12—N9	178.4 (2)
C3—N2—C4—N1	2.0 (3)	C9—N8—C12—N7	−1.1 (3)
C1—N1—C4—N3	176.72 (17)	C11—N7—C12—N9	−177.8 (2)
Cd1—N1—C4—N3	−16.7 (2)	C11—N7—C12—N8	1.7 (3)
C1—N1—C4—N2	−4.1 (2)		

Symmetry codes: (i) −*x*+1, −*y*+1, −*z*+1.

### Hydrogen-bond geometry (Å, °)

**Table:**

*D*—H···*A*	*D*—H	H···*A*	*D*···*A*	*D*—H···*A*
O1w—H11···N4	0.84 (1)	1.92 (1)	2.758 (2)	171 (3)
O1w—H12···N6^ii^	0.84 (1)	2.24 (1)	3.059 (3)	165 (2)
O2w—H21···N7	0.84 (1)	1.92 (1)	2.756 (2)	178 (3)
O2w—H22···O1	0.84 (1)	1.98 (1)	2.806 (2)	167 (3)
N3—H31···O2w^i^	0.85 (1)	2.28 (1)	3.070 (2)	154 (2)
N3—H32···N5^iii^	0.85 (1)	2.28 (1)	3.127 (2)	175 (2)
N6—H61···N2^iii^	0.85 (1)	2.23 (1)	3.071 (2)	173 (2)
N6—H62···O2^ii^	0.85 (1)	2.35 (1)	3.140 (2)	155 (2)
N9—H91···O3^iv^	0.85 (1)	2.20 (1)	3.009 (4)	159 (3)
N9—H92···O4	0.85 (1)	2.41 (2)	3.073 (3)	135 (3)

Symmetry codes: (ii) −*x*, −*y*+1, −*z*+1; (i) −*x*+1, −*y*+1, −*z*+1; (iii) −*x*, −*y*+1, −*z*; (iv) *x*, −*y*+3/2, *z*−1/2.
